# Modeling the Conductivity Response to NO_2_ Gas of Films Based on MWCNT Networks

**DOI:** 10.3390/s21144723

**Published:** 2021-07-10

**Authors:** Ada Fort, Marco Mugnaini, Enza Panzardi, Anna Lo Grasso, Ammar Al Hamry, Anurag Adiraju, Valerio Vignoli, Olfa Kanoun

**Affiliations:** 1 Department of Information Engineering and Mathematical Sciences, University of Siena, Via Roma 56, 53100 Siena, Italy; mugnaini@diism.unisi.it (M.M.); panzardi@diism.unisi.it (E.P.); alograsso@diism.unisi.it (A.L.G.); vignoli@diism.unisi.it (V.V.); 2 Chair Measurement and Sensor Technology, Department of Electrical Engineering and Information, Technology, Chemnitz University of Technology, 09107 Chemnitz, Germany; ammar.al-hamry@etit.tu-chemnitz.de (A.A.H.); adiraju.anurag@etit.tu-chemnitz.de (A.A.); Olfa.Kanoun@etit.tu-chemnitz.de (O.K.)

**Keywords:** MWCNT NO_2_ sensing, CNT gas sensing analytical model, gas sensing conductivity model, functionalized MWCNT, Au nanoparticles

## Abstract

This work proposes a model describing the dynamic behavior of sensing films based on functionalized MWCNT networks in terms of conductivity when exposed to time-variable concentrations of NO_2_ and operating with variable working temperatures. To test the proposed model, disordered networks of MWCNTs functionalized with COOH and Au nanoparticles were exploited. The model is derived from theoretical descriptions of the electronic transport in the nanotube network, of the NO_2_ chemisorption reaction and of the interaction of these two phenomena. The model is numerically implemented and then identified by estimating all the chemical/physical quantities involved and acting as parameters, through a model fitting procedure. Satisfactory results were obtained in the fitting process, and the identified model was used to further the analysis of the MWCNT sensing in dynamical conditions.

## 1. Introduction

The interest in using carbon nanotubes (CNTs) based materials has been continuously growing in the last years, due to their excellent physical, mechanical and electrical properties which make them suitable for various applications such as nano-optoelectronics, energy conversion systems, lithium-ion batteries production, supercapacitors, material composites and sensors, just to mention a few [1,2,3,4,5].

CNTs, as carbon-based material, present a hexagonal structure made of carbon atoms forming rolled-up sheets of graphite into a cylinder shape. According to the arrangement of the graphite sheets, two kinds of nanotubes can be identified: single-walled nanotubes (SWCNTs), which consist of a single layer of cylindrical graphene, and multiwall nanotubes (MWCNTs), which present multiple concentric layers [6,7]. The characteristic hollow shape and porous structure, the size, the large surface area and surface to volume ratio and the presence of defects, make CNTs a promising material for gas sensing layer realization [8,9,10].

Indeed, CNTs respond to adsorbates of different gas species, which change their electrical properties such as conductivity or permittivity, making CNTs a good solution for the realization of chemoresistive gas sensors, thus gaining a starring role alongside metal oxide (MOX) composites which are traditionally used in this field [11,12]. In particular, sensing films consisting of CNT disordered networks are gaining increasing popularity due to the facile preparation route and their good performance.

CNT networks act as semiconductor materials showing mainly a p-type behavior, and, in general, can respond both to oxidizing and reducing gases. Moreover, their particular structure makes them suitable for different techniques of functionalization, e.g., by means of the addition of nanostructured materials such as metal, metal oxides nanoparticles or organic polymers. This process can enhance the solubility and dispersion of the material and/or of speeding up the surface reaction between the target gas molecules and the CNTs. Finally, it can lead to an appreciable improvement of gas sensitivity and selectivity [13]. In particular, the functionalization by means of the carboxylic acid group (COOH) is used to improve the gas sensing performances of the pristine material since it can provide additional reactive sites on the side walls and at walls ends of the CNTs, improving the interaction with different analytes [9,14,15,16].

Among different CNTs, SWCNT showed better performances in terms of sensitivity to gas exposure with respect to the MWCNTs and this is mainly due to a lower percentage of semiconducting nanotubes that can be modulated by the target gas molecules, whereas the multi-wall structures show, in general, shorter response time and usually work at a lower temperature [17,18].

Both SWCNTs and MWCNTs showed an appreciable performance in the detection of several toxic and harmful gas species [19], and an extensive research activity has been carried out concerning nitrogen dioxide (NO_2_) detection.

In spite of the existing extensive literature on chemical resistive sensors based on CNTs, nowadays most knowledge on these devices is based on experimental characterizations with resulting unavoidable long-time procedures, due to test attempts and experimental error.

Indeed, in the literature many models are presented for gas sensors based on CNTs [20,21,22,23,24,25,26,27] but the majority of them treat single CNT devices (usually FETs) [22,23,24,25,26] or they do not arrive to an implementable mathematical model [27] and all those models are related to static conditions. A few models are developed for chemiresistors based on CNT networks [20,21], but these provide only a static description of their behavior, and the relationship between the transport mechanism and the chemical behavior is not based on a physical interpretation.

In this context, the need to develop a chemical/physical dynamic model aimed at predicting and/or simulating the gas sensing behavior of the material is of utmost importance, not only to speed up the development of the related measurement system but also to improve knowledge of the different physical and chemical mechanisms involved in sensing. Ad hoc simulation tools based on specific models permit to interpret the measured sensing behavior, because they allow the analysis of those quantities, which contribute to the sensing response but cannot be directly measured.

Following of the methodology followed by the authors in [28] and successfully applied to SWCNT [29], in this work we propose a model aimed at describing the conductivity of networks of MWCNTs when exposed to time-variable concentrations of NO_2_ and operating with variable working temperature. The model is derived from a theoretical description of the electronic transport in the nanotube networks, of the NO_2_ chemisorption reaction and of the interaction between these two phenomena. The model is numerically implemented and then identified by estimating all the chemical/physical quantities acting as parameters through the model fitting using specimens disordered networks consisting of MWCNTs functionalized with COOH groups, and with the addition of Au nanoparticles. The parameter estimation exploits dynamical measurements obtained with time-variant temperature and NO_2_ concentration. The paper provides a procedure of general validity, which enables other researchers to obtain a simulation tool for similar sensors.

The paper presents the derivation and validation of a novel model for chemoresistive sensors based on disordered MWCNT networks while also describing their dynamic behavior both in case of variable operating temperature and chemical environment. This type of model, to the authors’ knowledge, does not exist. The model links the kinetics of the chemical reactions at the surface of the film with electronic conduction.

## 2. Model Derivation

### 2.1. Theoretical Derivation

MWCNTs used for gas sensing applications are semiconductors with a band-gap depending on their molecular structure [8,30,31] in which unintentional defects act as acceptor dopants [31] creating holes, such that MWCNT networks usually behave as p-type semiconductors. In this section we present a model describing the conductivity of networks of MWCNTs, obtained with the same approach used for SWCNT networks by the authors in [29]. The final aim of the model is to get a relationship between the film resistance and both the working temperature and NO_2_ concentration in dynamic conditions. Therefore, the chemical/physical model requires two steps: modeling the kinetics of the interaction of the gas with the material under test and describing the effect that this interaction has on electronic conduction. As far as the first step is concerned, regarding performing NO_2_ sensing in air mixtures, the searched conductivity model is related to the reversible chemisorption of both NO_2_ and O_2_ on the nanotube walls, described by the following reactions [29]:

(1) $$\frac{1}{2}O_{2_{gas}}+S\begin{array}{c} \overset{k_{O}}{\to }\\ \underset{k_{iO}}{\leftarrow } \end{array}\left(O^{-}-S\right)$$

and:

(2) $$NO_{2}{}_{gas}+S\begin{array}{c} \overset{k_{NO2}}{\to }\\ \underset{k_{iNO2}}{\leftarrow } \end{array}\left(NO_{2}^{-}-S\right)$$

where *S* is an adsorption site whereas *k_Y_* and *k_iY_* indicate the adsorption/desorption rate constants for the species *Y*. The reaction rate constants can be expressed in an Arrhenius form of the type:

(3) $$k_{Y}=k_{0Y}exp\;\left(-\frac{\lambda_{Y}}{T}\right)\;;\;k_{iY}=k_{0iY}exp\;\left(-\frac{\lambda_{iY}}{T}\right)\;$$

where *k*_0*Y*_ and *k*_0*iY*_ are the pre-exponential terms, whereas the quantities $\lambda_{Y}$ and $\lambda_{iY}\;$ are temperatures, related to the activation energies of adsorption/desorption of the species *Y*, *E_Y_*_,_ and $\lambda_{Y}=\frac{E_{Y}}{k}$, with *k* being the Boltzmann constant. From now on, the possible weak dependences on the temperature of all the pre-exponential terms, *k*_0*Y*_ and *k*_0*iY*_, are neglected.

For brevity’s sake, in the following, we will use the undermentioned symbols:

$$\left[O^{-}-S\right]=N_{0}\;\mathrm{and}\;\left[\;NO_{2}^{-}-S\right]=N_{NO2}$$

where [*Y*] is the surface density of the adsorbed species *Y*.

Therefore, the surface density of the adsorbed negative charge is *Ns* = *N*_0_ + *N_NO_*_2_.

The kinetics of the two surface reactions can be described by the following differential equations:

(4) $$\frac{dN_{O}}{dt}=k_{O}{\left({\left[O_{2}\right]}_{gas}\right)}^{\frac{1}{2}}\left(\left[S\right]-N_{O}-N_{NO2}\right)-k_{iO}N_{O}$$

(5) $$\frac{dN_{NO2}}{dt}=k_{NO2}{\left[NO_{2}\right]}_{gas}\left(\left[S\right]-N_{O}-N_{NO2}\right)-k_{iNO2}N_{NO2}$$

where [*Z*] denotes the concentration of the gas *Z*, whereas [*S*] is the surface density of the adsorption sites.

Equations (4) and (5) describe two first order reactions accounting for ionization as the rate limiting step. If we take into account the Arrhenius form reported in Equation (3), Equation (4) can be rearranged as follows:

(6) $$\frac{dN_{O}}{dt}=A_{0}k_{1}e^{-\frac{\lambda_{o_{}}}{T}}\left(\left[S\right]-N_{O}-N_{NO2}\right)-k_{0iO_{}}e^{-\frac{\lambda_{iO_{}}}{T}}N_{O}$$

(7) $$\frac{dN_{NO2}}{dt}=A_{NO_{2}}k_{2}e^{-\frac{\lambda_{NO}{}_{2}}{T}}\left(\left[S\right]-N_{O}-N_{NO2}\right)-k_{0iNO_{2}}e^{-\frac{\lambda i_{NO}{}_{2}}{T}}N_{NO2}$$

where the quantities *A*_0_, *A_NO_*_2_, $k_{1}$ and $\;k_{2}$ are constant and can be defined as follows:

(8) $$A_{0}=\frac{{\left({\left[O_{2}\right]}_{gas}\right)}^{\frac{1}{2}}}{{\left({\left[O_{2}\right]}_{gas}^{REF}\right)}^{\frac{1}{2}}};\;A_{NO_{2}}=\frac{{\left[NO_{2}\right]}_{gas}}{{\left[NO_{2}\right]}_{gas}^{REF}}\;$$

whereas:

(9) $$k_{1}=k_{0O}{\left({\left[O_{2}\right]}_{gas}^{REF}\right)}^{\frac{1}{2}}\;;\;k_{2}=k_{0NO_{2}}{\left[NO_{2}\right]}_{gas}^{REF}\;$$

with ${\left[O_{2}\right]}_{gas}^{REF}$ and ${\left[NO_{2}\right]}_{gas}^{REF}$ being arbitrary reference gas concentrations.

In this work, neither the gas concentrations nor the temperature are assumed as constant quantities, therefore Equation (4) and (5) will be used to describe the variation with time of the surface density of adsorbed charge due to temperature or to gas concentration variations. Therefore, considering *T* = *T*(*t*), *A_O_ = A_O_*(*t*) and *A_NO_*_2_ = *A_NO_*_2_(*t*)*,* the time dependent surface densities can be found solving two linear differential equations with time-variant coefficients defined by nine parameters ($k_{1},k_{2},k_{0iO},k_{0iNO_{2}},\lambda_{O},\lambda_{NO_{2}},\lambda_{iO},\;\lambda_{iNO_{2}},\;$ [*S*]) that describe the gas–nanotube interaction. In particular, the constant parameters depend on the sensing material characteristics. Different values must be used for the pure MWCNTs–COOH and for those functionalized by the Au nanoparticles which will act as a catalyst and favor the adsorption of the target gas [28].

To obtain the complete model for the sensing film resistance, it is necessary to describe how these quantities affect the resistivity of the films. To this end the electronic transport mechanism must be explained. In this paper we consider sensing films consisting of disordered networks of MWCNTs. For this kind of materials, in analogy to what was also found for SWCNTs [29,32], the conduction is due to the intra tube and on the inter-tube carrier transport. In particular, the conduction across the junctions between two nanotubes can be described by fluctuation-assisted tunneling across the inter-tube barriers [20,33], and as such is a temperature activated conduction, similar to the one observed in semiconductor materials. On the other hand, the intra-tube transport is hindered at high temperature due to carrier scattering caused mainly by wall defects; therefore, due to its dependence on temperature, it can be seen as a “metallic contribution”. The intra-tube transport becomes more important at high temperatures due to the increased probability of tunneling across the barriers. In particular, according to the literature, the resistivity, $\rho \left(T\right)$, of an MWCNT network can be related to the working temperature, *T*, as follows [34]:

(10) $$\rho \left(T\right)=\rho_{so}exp\;\left(\frac{T_{1}}{T_{0}+T}\right)+\rho_{m0}exp\;\left(-\frac{T_{m}}{T}\right)\;$$

where $\rho_{so}$ and $\rho_{m0}$ are quantities that can be considered constant with respect to temperature, *T_m_* is a characteristic temperature defining the “metallic behavior”, whereas *T*_1_ and *T*_0_ are described by the following equations:

(11) $$T_{1}=\alpha \epsilon_{0}\;\frac{8A}{w}{\left(\frac{U}{q\;}\right)}^{2}=\beta^{2}U^{2}$$

(12) $$T_{0}=T_{1}\frac{h}{\pi^{2}w}\frac{1}{\sqrt{2mU}}$$

In Equations (11) and (12), *A* and *w* are the area and the width of the inter-tube junction, *h* is the Planck constant, ε_0_ is the vacuum permittivity, *q* is the electron charge, *m* is the free carrier effective mass, α is a constant depending on the molecule and, finally, *U* is the potential barrier height at the inter-tube junction. In the rightmost term of Equation (11) we define the quantity $\beta =\frac{1}{q}\sqrt{\frac{\alpha \epsilon_{0}8A}{w}}$ which can be considered a constant value.

Therefore, from now on, the film conductance, *R*, will be described as follows:

(13) $$R=R_{B}exp\;\left(\frac{T_{1}}{T_{0}+T}\right)+R_{A}exp\;\left(-\frac{T_{m}}{T}\right)\;$$

where $R_{B}$ and $R_{A}$ are parameters depending on the film geometry, on $\rho_{m0}\;$ and $\rho_{so}$ in Equation (10), and as such are assumed independent of temperature.

Moreover, exploiting Equation (12), we can write:

(14) $$R=R_{B}exp\left(\frac{1}{\frac{\delta \sqrt{\beta }}{\sqrt[{4}]{T_{1}}}+\frac{T}{T_{1}}}\right)+R_{A}exp\left(-\frac{T_{m}}{T}\right)=R_{B}exp\left(\frac{T_{1}}{\nu T_{1}^{3/4}+T}\right)+R_{A}exp\;\left(-\frac{T_{m}}{T}\right)\;$$

where $\delta =\frac{h}{\pi^{2}w}\frac{1}{\sqrt{2m}}$ and $U=\frac{\sqrt{T_{1}}}{\beta }$.

From Equation (14) it can be seen that the electrical modeling of the film requires four further parameters, i.e., ν, $R_{B},\;\;R_{A}$ and *T_m_* depending on the sensing material characteristics, on the sensing film geometry and on the quantity *T*_1_, depending in turn on the height of the potential barrier at the grain boundary, which is assumed to vary in different working conditions of the sensing film.

Summarizing, Equation (14) provides the relationship between the resistance and the working temperature explicitly, but to obtain a complete model for the dynamic response of MWCNT networks to NO_2_, *T*_1_, which determines the tunneling probability, has to be related to the target gas concentration through the adsorbed gas surface density.

In this paper, we accept the same assumption used in [29], which was verified by experimental results for SWCNTs, i.e., that the height of the potential barrier, *U*, at inter-tube junction decreases linearly as a function of the adsorbed charge surface density through a sensitivity factor *γ*. Therefore, we assume:

(15) $$U=U_{0}-\gamma N_{s}=U_{0}-\gamma \left(N_{O}+N_{NO2}\right)$$

Equation (15) describes how the probability of tunneling is favored by the adsorbates and, from this equation, the dependence of the quantity *T*_1_ on *N_S_* can be explicated as follows:

(16) $$T_{1}=\beta^{2}{\left(U_{0}-\gamma N_{s}\right)}^{2}=\beta^{2}\left(U_{0}^{2}-2U_{0}\gamma N_{S}+\gamma^{2}N_{s}^{2}\right)=T_{10}-2\sqrt{T_{10}}\;\beta \gamma N_{S}^{}+\beta^{2}\gamma^{2}N_{S}^{2}$$

where *T*_10_ represents the value of *T*_1_ in the absence of any adsorbate.

Equation (16) can be replaced in Equation (14), to obtain the searched result.

Moreover, it is found from Equation (16) that *T*_1_ can be simply derived from a scaled version of the density of adsorbed species $N_{s}^{\prime }=\gamma \beta N_{s}$, as follows:

(17) $$T_{1}=T_{10}-2\sqrt{T_{10}}\;N_{S}^{\prime }+N_{S}^{\prime 2}$$

Therefore, *T*_1_ can be calculated as a function of time, temperature and gas concentration, starting from *N_s_*′ and avoiding the estimation of any additional parameter, by numerically solving the differential Equation (6) and (7) rewritten for $N_{O}^{\prime }=\gamma \beta N_{O};\;N_{NO2}^{\prime }=\gamma \beta N_{NO2}$ as:

(18) $$\frac{dN_{O}^{\prime }}{dt}=A_{0}k_{1}e^{-\frac{\lambda_{O}}{T}}\left({\left[S\right]}^{\prime }-N_{O}^{\prime }-N\prime {}_{NO2}\right)-k_{0iO}e^{-\frac{\lambda_{iO}}{T}}N_{O}^{\prime }$$

(19) $$\frac{dN_{NO2}^{\prime }}{dt}=A_{NO_{2}}k_{2}e^{-\frac{\lambda_{NO}{}_{2}}{T}}\left({\left[S\right]}^{\prime }-N_{O}^{\prime }-N\prime_{NO2}\right)-k_{0iNO_{2}}e^{-\frac{\lambda i_{NO}{}_{2}}{T}}N_{NO2}^{\prime }$$

where [*S*]′ = *γβ*[*S*].

### 2.2. Model Calibration Procedure (Parameters Estimation)

**Figure 1:**
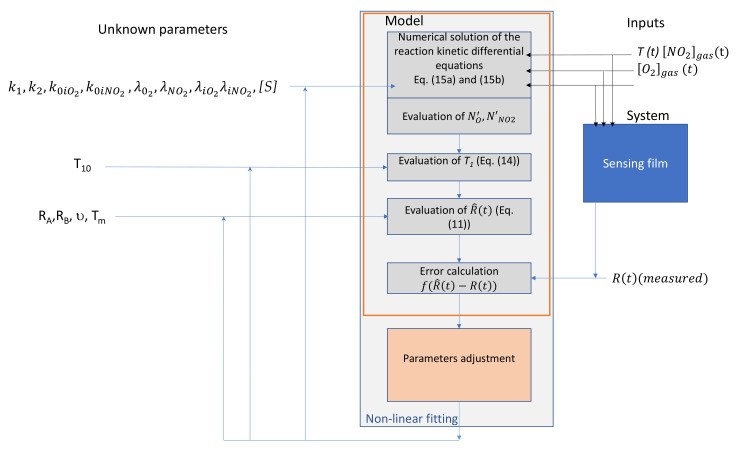
Parameter estimation procedure.

The parameters used in the theoretical model (listed in Figure 1) are unknown, and they critically depend on the particular material and device used for the experiments. Therefore, they cannot be derived from the literature.

For instance, it is well known that the rate constants of the chemisorption reactions depend dramatically on defects which in turn are created due to the particular preparation route and material pre-treatment.

In this paper, the estimation of these parameters, described in Figure 1, is treated as a classical system identification problem. The model structure is fixed and coincides with the one theoretically derived in the previous subsection. Parameter identification is performed to obtain the coefficients in the functional system exploiting measurement data. The approach followed by the authors in this and other papers [29,35] is to carry out experiments on the system to collect data, through which the needed parameters are then estimated. It is known that, to achieve experimental identifiability, the dataset must be «sufficiently informative» concerning the parameters of interest. Therefore, the input signals used to excite the system (temperature and gas concentration) must ensure experimental identifiability (so they must be «exciting» in a suitable sense). In Figure 1 the identification procedure is described in detail. There, it is summarized that the model’s inputs are the three dynamical signals: working temperature and gas concentrations (NO_2_ and oxygen). To summarize, to obtain a reliable parameter estimation, it is essential to select a measurement protocol ensuring large and fast variations of all the input signals in order to excite the different dynamics involved in the sensing film response to NO_2_.

The developed model was numerically implemented in Matlab, minimizing the number of parameters involved according to the procedure described in the Model Derivation section and used to fit the experimental data. The differential Equations (18) and (19) were numerically solved using the forward Euler method.

A non-linear least-square fitting (lsqnonlin) based on the Levenberg–Marquardt algorithm was used to adjust the model’s unknown parameters aiming at minimizing the error function which is the root mean square value of the relative error between the predicted and the measured resistance or conductance. The choice of the initial values of the parameters is critical. The method used to cope with this problem will be described in detail in the following, and it is based on the use of independent measurements and the analysis of the experimental data.

## 3. Materials and Methods

### 3.1. Materials

MWCNTs functionalized with COOH group (2.5 wt%) were purchased in powder form from NanoAmor Inc. The MWCNTs have a purity >95% and present an external diameter in the range of 8 nm–15 nm with a length of 0.5 µm–2 µm. An isopropanol-based solution was obtained by dispersing 0.05% of the MWCNTs (wt%) and sonicating with a spherical tip for 90 min. A sonication power of 30 W was applied with square pulses (50% duty cycle). The sonication time was chosen after time optimization and used for the rest of the investigation.

The Au nanoparticles used for the sensing film functionalization were purchased by Sigma Aldrich (Darmstadt, Germany). The material was prepared in a phosphate-buffered saline (PBS) suspended solution with concentration of 0.1 mM. The nanoparticles have a declared average size of 5 nm in diameter and 5.5 × 10^13^ particles/mL concentration with a molar mass of 196.97 g/mol, and a declared variability of less than 12% in size and shape.

### 3.2. Sensing Film Preparation

**Figure 2:**
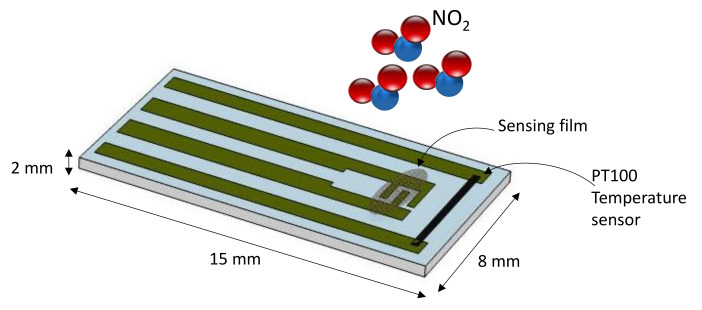
Sensor structure.

The sensing films, based on MWCNT networks and used to test the applicability of the proposed model, were deposited on an alumina substrate equipped with screen-printed silver electrodes and a platinum resistive temperature sensor (Pt-RTD) (as described in [28,36]). A heater was realized on the back side of the substrate with the same technique. The size and the structure of the device are shown in Figure 2. As it can be seen, this structure allows for testing the behavior of the material at a temperature measured with high accuracy, since the temperature sensor is deposited on the same substrate and very close to the sensing film.

Before the deposition of the sensing layer, the pristine substrates were cleaned with ultrapure water and then heated in the oven at 400 °C for two hours, in order to remove any potential impurities. The MWCNT network layer was obtained by drop-casting 2 μL of the base MWCNTs solution, described in the previous subsection, on the alumina substrate within the interdigitated electrodes, dried at room temperature and then heated at 280 °C for two hours. The functionalization with Au nanoparticles was obtained by two consecutive depositions of 1 μL of the Au suspended solution on the obtained MWCNT based films (corresponding to about a 40 ng Au mass). The functionalized samples were dried at room temperature and subsequently heated at 280 °C for 4 h.

### 3.3. Sensing Material Characterization

UV–Vis–NIR spectroscopy was used to evaluate the dispersion of the MWCNT in the prepared solution and to optimize the preparation method. In particular, the UV–Vis spectrometer Cary 60 from Agilent was used to measure the absorption spectra of the prepared dispersions. For the UV–vis–NIR analysis, the original MWCNT–COOH solution was diluted by mixing 30 µL (100×), 60 µL (50×) and 90 µL (25×) in 3 mL of isopropanol and then deposited by dip-coating on glass substrates and let dry at room temperature.

To evaluate the morphology of the tested sensing films, an atomic force microscopy (AFM) analysis was used. In particular, images of films obtained with the sensing materials described in the previous subsections were carried out by the Keysight 5600L AFM equipment. Two different samples were prepared on glass substrates for the investigation: one was obtained by drop casting the sensing solution (as for the sensing film preparation), whereas the other one was realized by dip-coating and quickly cleaning the substrate, in a way to leave just some of the material and to allow the observation of individual MWCNT structures.

Finally, the stability of the material vs. temperature was tested through Raman spectroscopy. Raman spectra were obtained by Horiba Xplora instrument at a wavelength of 532 nm in 2 min of acquisition time. For Raman measurements, samples were prepared by drop-casting 10 µL of the MWCNT–COOH based solution on a silicon substrate, and dried at room temperature (as for the sensing film preparation). Measurements were taken after heating the samples for 20 min at different temperatures, i.e., 140 °C, and 240 °C.

### 3.4. Impedance Characterization

The impedance of the realized device was measured in static conditions by means of a commercial impedance analyzer (Waine Kerr 6500B), in the frequency range 5 Hz–2 MHz. The impedance spectra were measured in controlled working conditions, with constant and different temperatures and gas composition environments.

### 3.5. Gas Sensing Properties Characterization System

**Figure 3:**
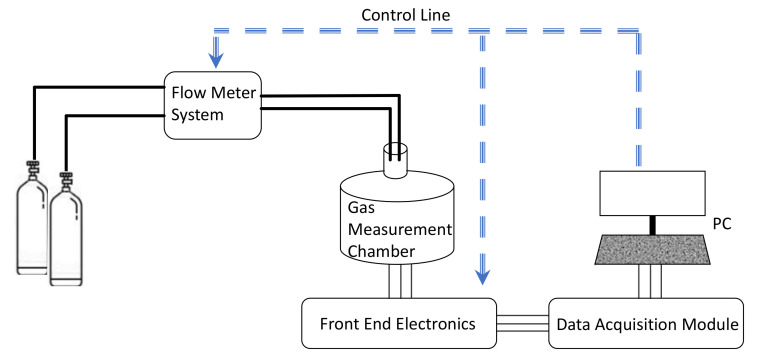
Schematic representation of the characterization system.

The gas sensing layer conductance in dynamical conditions was measured using an automated measurement and characterization system, described in detail by the authors in [37] and schematically depicted in Figure 3. The system provides the possibility to acquire and evaluate the conductance of the sensors under test in real-time and for different measurement conditions. In particular, the system allows to remotely manage the concentration of the gas mixture and the working temperature. The total flow of the gas in the measurement chamber was kept constant during all the measurements, and it was set to 200 mL/min, whereas its composition was varied by means of digitally controlled flow meters (BronkHorst F-201C). The working temperature, that can be controlled and varied during measurements thanks to a feedback control system which exploits the temperature measured through the Pt-RTD sensor placed on each of the tested samples, was set in the range 120–220 °C, with a resolution of approximately 1 °C.

## 4. Characterization Results

### 4.1. Material Characterization

**Figure 4:**
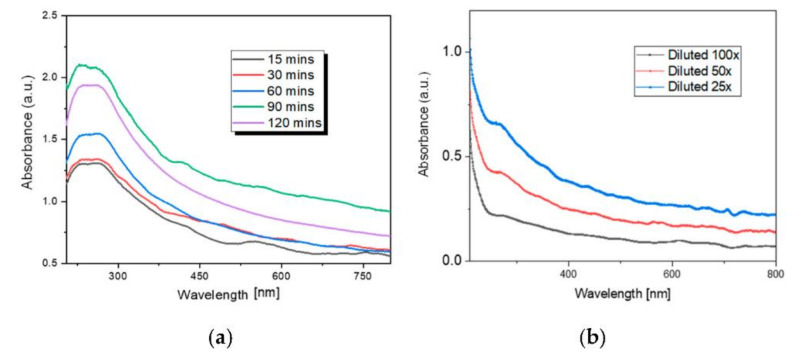
UV–Vis spectra of MWCNT–COOH isopropanol dispersed solution: (**a**) different dilutions; (**b**) different sonication times for the 25× diluted sample.

Figure 4a reports the obtained results from UV–Vis spectrometry for the three different dilutions, 100×, 50× and 25× (see Section 3.3), whereas, in Figure 4b the spectra of the selected 25× dilution taken at different sonication times are reported. For all the tested cases, the peak is at about 250 nm and the spectra amplitude gradually reduces as the wavelength increases. The absorbance of the dispersion increases with the sonication time up to 90 min, after that the agglomeration of MWCNTs reduces its absorption capability. Based on the UV–Vis measurements results, the solution sonicated for 90 min was used for the sensing film deposition and for the following analyses.

**Figure 5:**
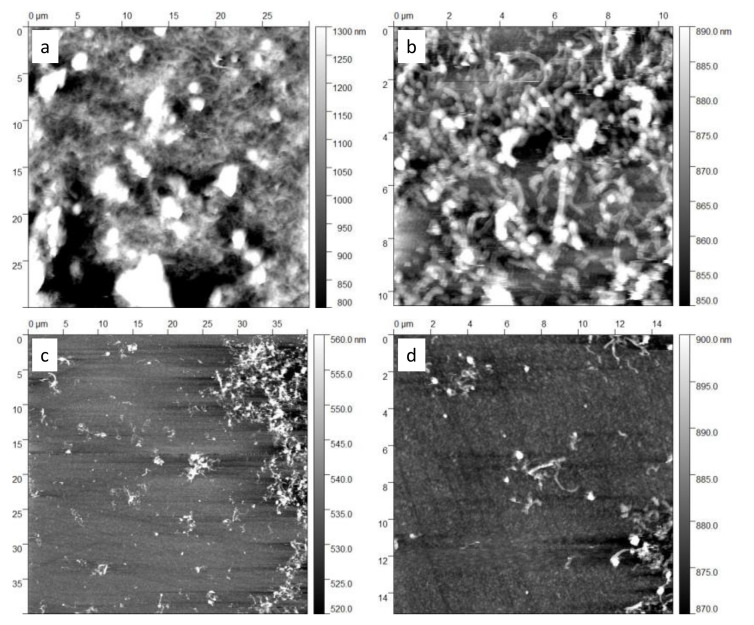
Atomic force microscopy (AFM) images of two MWCNT–COOH films on a glass substrate: drop-casted film (**a**,**b**); dip-coated film (**c**,**d**). Images (**b**,**d**) are detail magnifications.

**Figure 6:**
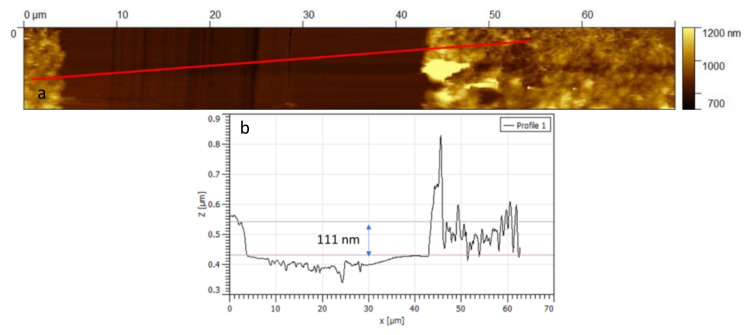
Atomic force microscopy (AFM) images of 2 µL of the MWCNT–COOH solution drop-casted on a glass substrate (**a**) and evaluated film thickness (**b**).

Figure 5a,b show some AFM images of the drop-casted film where the formation of a continuous film and the presence of MWCNT networks with a rather homogeneous distribution can be observed (roughness in Figure 5b is 12 nm). The presence of large agglomerates can be noted, that probably were formed due to the fast evaporation of isopropanol after the deposition on the substrate. In Figure 5c,d, images of the dip-coated sample are reported (roughness in Figure 5c is 7 nm), where individual structures of CNTs can be observed, confirming the expected sizes without significant changes or shortening effects due to the dispersion procedure. Moreover, the thickness of the deposited film was measured by AFM analysis, as shown in Figure 6, and a value of approximately 110 nm was estimated.

**Figure 7:**
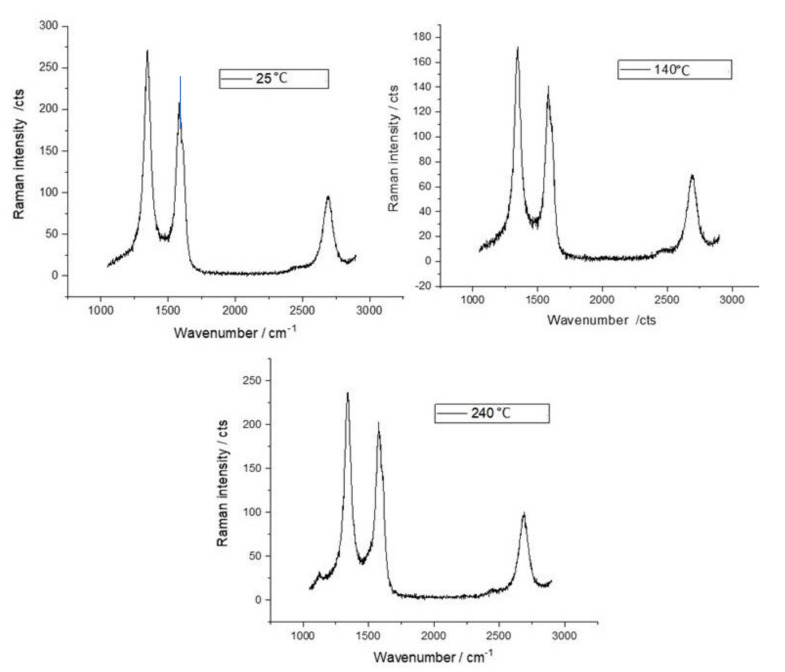
Raman spectra of the MWCNT–COOH solution drop-casted on a silicon substrate and heated at different temperatures as per legend.

Figure 7 shows the Raman spectra of the selected MWCNTs based solution, which was the one sonicated for 90 min. As it can be seen from the spectra, the D peak is around 1340 cm^−1^, the G peak is at 1570 cm^−1^ and the 2D peak is at about 2700 cm^−1^. The D peak is due to the defects, the G peak resembles the graphitic nature of the sample, whereas the 2D peak arises due to the two phonon second order scattering process [38]. The results show that the peaks’ positions are not altered as the temperature increases and that the intensity of the G band increases as the temperature rises to 140 °C; accordingly, the I_D_/I_G_ ratio decreases from 1.29 at room temperature to 1.14. This could be termed as a “low defect” density region [38], wherein the I_D_/I_G_ ratio decreases with the increase in defect intensity, as increase in G peak is more appreciable than D peak. The ratio remains rather steady up to 240 °C, when it starts to increase reaching a value of 1.26. This could be termed as a “high defect” density region [38], where the increase in defect density dominates the sp^2^ nature of the sample (G peak). This could be attributed to the removal of the functional groups from the surface leading to asymmetry in the structure [39].

These results show that this material is stable and can be heated for experiments up to 240 °C.

Note that, in this work, we used the same Au NPs and the same preparation and functionalization procedures for the sensing film as in [28], in which, through SEM, TEM and elemental analyses, we could infer that metal Au NPs form clusters evenly distributed on the CNT network.

### 4.2. Electrical Characterization

The characterization of the electrical behavior of the devices described in the previous section was performed through impedance spectrum analysis to investigate the role of the electrode film/interface in the overall impedance. In particular, measurements aimed to ascertain that the conductance measurements could be used to validate the proposed model of the sensing film, since they are not affected by the contribution of the electrode/film interfaces.

**Figure 8:**
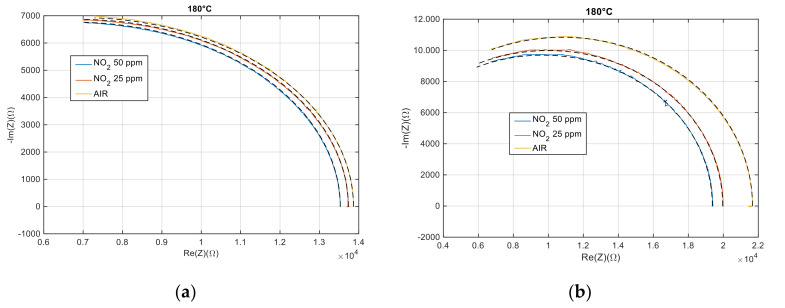
Impedance measurements performed in the frequency range 5 Hz–20 MHz represented in the complex plane. Each curve represents a measurement obtained in stationary conditions at the fixed working temperature of 180 °C, under a fixed gas flow (200 mL/min) and with a fixed NO_2_ concentration. The carrier gas was dry air; (**a**) MWCNT–COOH, (**b**) MWCNT–COOH and Au nanoparticles.

The impedance spectra analysis pointed out that, in the considered working conditions, the tested devices act as resistances with small fixed parasitic capacitances (of about 6 pF) in parallel, indicating that the electrodes/film interfaces provide negligible contributions. Figure 8 shows, as examples, the measured impedances in the complex plane, in dry air and in the presence of different NO_2_ concentrations at the fixed and controlled temperature of 180° for the pure MWCNT–COOH based device (Figure 8a) and of the one obtained by Au nanoparticle functionalization (Figure 8b). The obtained results show that the developed devices can be used to model the electrical behavior of the sensing film as desired.

**Figure 9:**
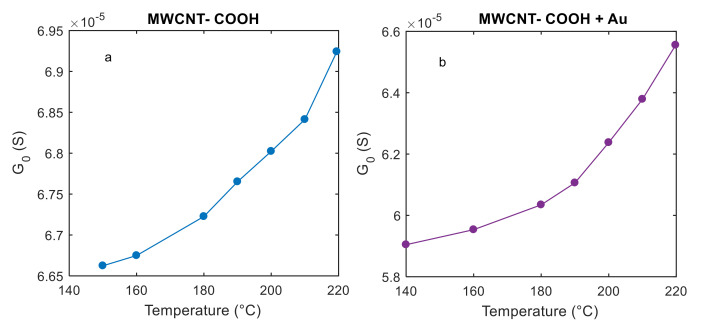
Steady state conductance (G_0_) of the tested films in air as a function of working temperature (constant). The measurements were performed under constant dry air gas flow of 200 mL/and at a constant temperature. (**a**) MWCNT–COOH; (**b**) MWCNT–COOH and Au nanoparticles.

A further characterization was carried out to evaluate the temperature dependence of the baseline conductance (G_0_) of the tested materials in air. The results obtained for one of the films before and after the Au functionalization are shown in Figure 9. According to the results obtained from the material characterization, the selected temperature range does not exceed 220 °C in this work.

As shown in Figure 9, the measured conductance values increase with temperature in the range considered in this paper (*T* ≤ 220 °C), this suggests that the contribution of the metallic term in Equation (14) could give a negligible contribution, and that, as a first attempt, a simplified model for the conductance can be used to fit data. This result is consistent with other findings in the literature, e.g., [34]. Under this assumption, the film conductance could be described by the following equation:

(20) $$G={\frac{1}{R_{B}}}_{}exp\left(-\frac{T_{1}}{\nu T_{1}^{3/4}+T}\right)\;.$$

Note that when describing the film conductance with this equation the overall resistance of the film is made up of parallel and series of resistances of the type $R_{0n}exp\;\left(\frac{T_{1}}{T_{0}+T}\right)$ related to a single barrier at an inter-tube boundary. The morphology and the geometry of the film influence the number of series and parallel elements and, as such, they determine the value of the pre-exponential factor *R_B_* in Equation (13) or in Equation (20). Note that, if the thickness of the film remains small (in the thin film range), and the dynamic of the gas diffusion into the film remains fast with respect to the one of chemisorption, the effect of thickness variation can be neglected in the derived dynamical model. This is the case of the tested films, for which AFM analysis confirmed a thickness of the order of 100 nm.

Therefore, in the proposed model the variability of the film (size and thickness) translates into a different value of baseline conductance.

## 5. Model Parameter Estimation: Experimental Results and Discussion

In this section the results of model calibration, i.e., the parameter estimation will be presented. The obtained results can be used to further the knowledge of the sensing mechanism and to analyze the performance of the tested materials and of materials (such as other disordered networks) whose conduction mechanism is based on the same phenomenon.

The parameter estimation was obtained adopting the method illustrated in Section 2.2, exploiting the measurements obtained exciting the sensing films under test by means of different stimuli consisting of variable temperatures and gas concentrations.

The temperature input signal was actuated using the closed-loop temperature control system embedding the Pt-sensor located close to the film on the same substrate, as described in Section 3, which provides an accurate measurement of this signal. On the other hand, the gas concentration input is derived from the flowmeter settings.

The measurement protocol selected for the estimation is based on gas concentration pulses, superimposed to temperature pulses. The time lengths of the pulses and their amplitudes were selected based on the expected thermal dynamics of the device and typical chemical reaction kinetics.

The results discussed in the previous section show that the metallic contribution is not the dominant term. Therefore, in the following, the simplified model of the sensing film reported in Equation (20) will be used, in which only the effect of the tunneling across the inter-tube barriers is accounted for.

Unfortunately, even the simplified version of the model needs the estimation of a large number of parameters ($k_{1},k_{2},k_{0iO},k_{0iNO_{2}},\;\lambda_{O},\lambda_{NO_{2}},\lambda_{iO},\;\lambda_{iNO_{2}},\;$ [*S*], $R_{B},\;\nu ,\;T_{10}$), and this is a known criticality that can lead to the identification of models able to fit well the data used for calibration but unable to generalize, i.e., it can provide unreliable fitting results.

Therefore, the number of parameters to be estimated in a single identification step, i.e., exploiting the same measurements, was conveniently reduced using information based on independent and different experiments, following the approach adopted by the authors in other works [29].

First of all, experiments in air in the absence of NO_2_ were planned to obtain the subset of parameters ($k_{1},k_{0iO},\lambda_{O},\lambda_{iO},\;$ [*S*], $R_{B},\;\nu ,\;T_{10}$) that are related, as far as the chemical reactions are concerned, only to the chemosorption of oxygen.

**Figure 10:**
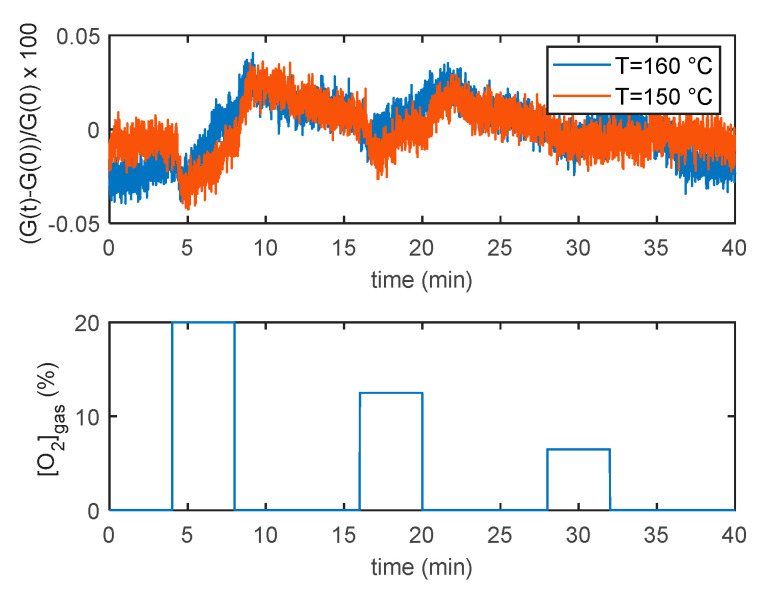
Response to variation of oxygen gas concentration (gas concentration profile is reported in the lower plot) for the MWCNT–COOH film at two fixed working temperatures. The film response is defined in the upper plot *y*-axes label as the normalized conductance variation ((G(t)−G(0))/G(0)) in percentage.

The experiments were performed varying the concentration of oxygen, by mixing synthetic air and nitrogen, and led to the conclusion that, for the tested material, the chemisorption of oxygen has a negligible effect on conductivity. An example of experimental results supporting this conclusion is shown in Figure 10, where the responses of the pristine MWCNT–COOH film to large variations of the oxygen concentration at two fixed temperatures are shown. The relative variation of conductivity due to a variation from 20% of oxygen to 0% is below 0.1% in the whole tested temperature range, and can be considered comparable to perturbations due to flow fluctuations and other disturbances. The Au functionalized film shows even smaller responses that cannot be distinguished from the conductance baseline’s smaller fluctuations.

This result allows for further simplifying the model, neglecting the contribution of oxygen, and reducing the parameter set to $(k_{2},k_{0iNO_{2}}{}_{},\;\lambda_{NO_{2}},\lambda_{iNO_{2}},\;$ [*S*], $R_{B},\;\nu ,\;T_{10}$).

**Figure 11:**
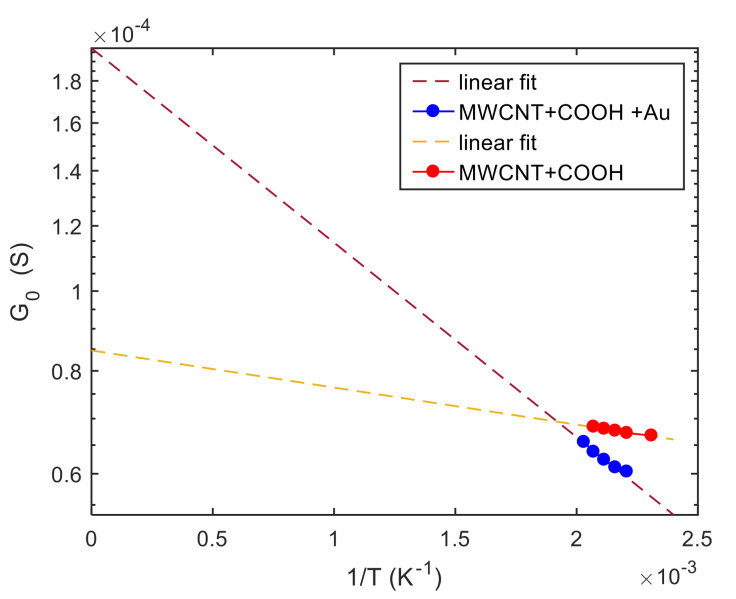
Linear fit of log(*G*_0_), *G*_0_ is the film conductance in air, at constant temperature (*T*) as a function of 1/*T*.

The number of parameters is still critical, and to help the fitting procedure, a first raw estimation of the “electrical” parameters $R_{B}\;\mathrm{and}\;T_{10}$ was derived from the electrical characterization presented in the previous section. In particular, raw estimations of *R_B_* and *T*_10_ were found from the *G*_0_ vs. *T* measurements in air, presented in Figure 11, based on the fact that, in air, the potential barrier height at the inter-tube boundaries is almost constant since $N_{s}^{\prime }\approx 0$: therefore $T_{1}\approx T_{10}$ considering $\nu T_{10}^{3/4}\ll T$ in Equation (20). In this case, we have:

(21) $$\log G_{0}\approx -\log\left(R_{B}\right)-\frac{T_{10}}{T}$$

The two searched parameters were found from the linear regression, shown in Figure 11 as the slope and intercept of the fitting lines.

Note that the parameters estimated by the fitting in Figure 11 can be affected by large errors due to the small temperature ranges and to the approximations in Equation (21), this is the reason why we used these raw estimations as parameter initial values in the fitting algorithm.

**Figure 12:**
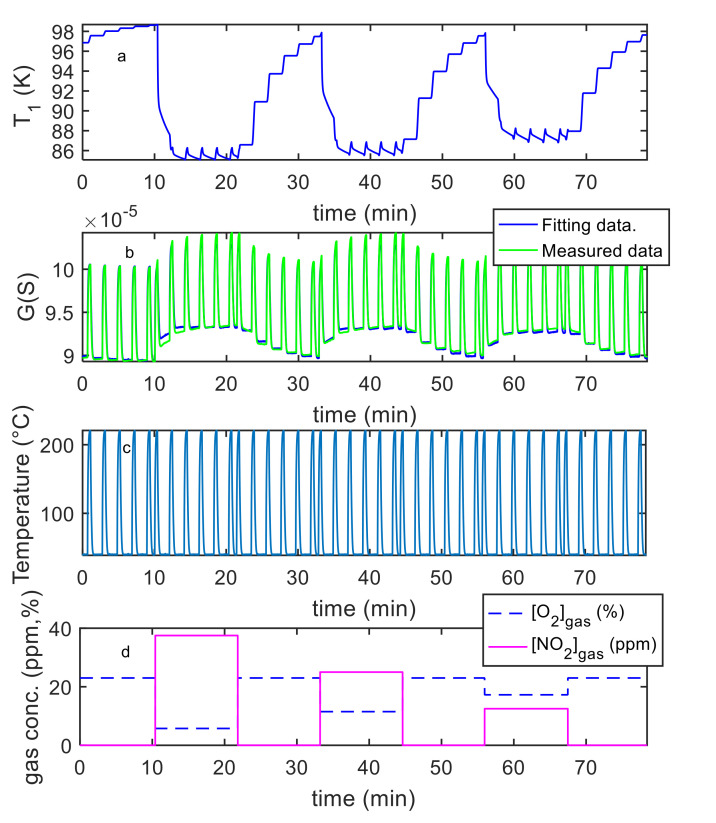
Results of the model fitting for an MWCNT–COOH sensing film. From the top: the first plot (**a**) shows the estimated *T*_1_ as a function of time (model output), the second plot (**b**) compares the estimated *G*(*t*) (model output) with the measured one as per legend, the third (**c**) shows the measured temperature (model input), the last (**d**) the gas concentrations (model inputs).

**Figure 13:**
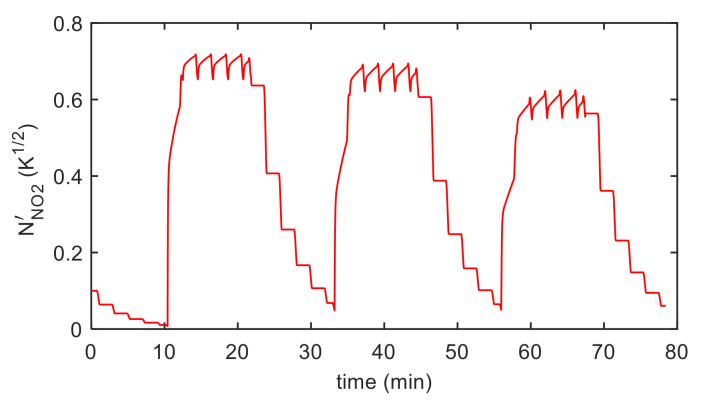
Results of the model fitting for a MWCN–COOH sensing film. Estimated chemisorbed NO_2_ as a function of time (model output).

**Figure 14:**
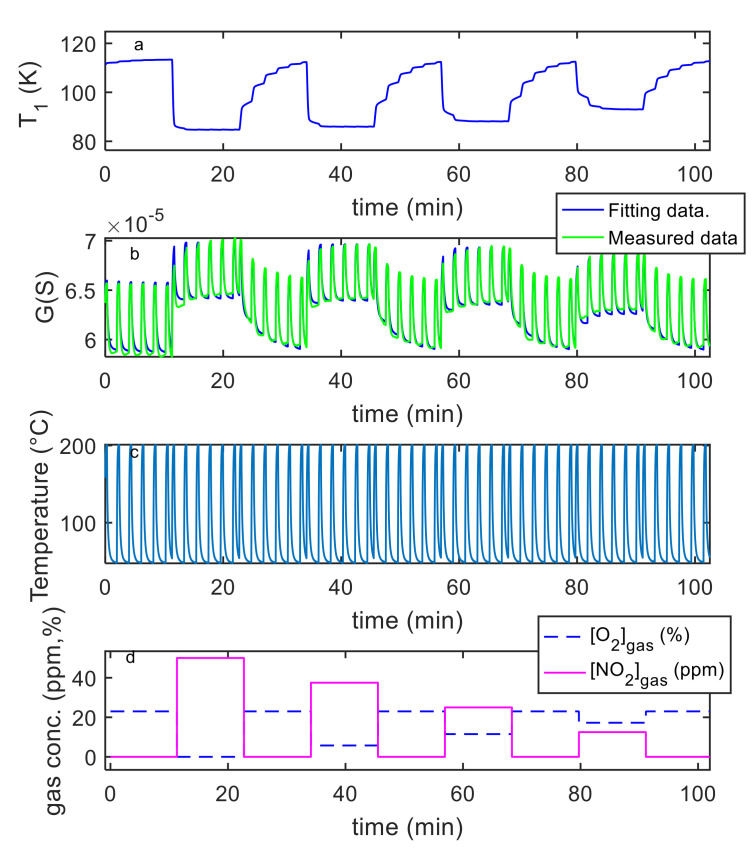
Results of the model fitting for an Au decorated MWCNT–COOH sensing film. From the top: the first plot (**a**) shows the estimated *T*_1_ as a function of time (model output), the second plot (**b**) compares the estimated *G*(*t*) (model output) with the measured one as per legend, the third (**c**) shows the measured temperature (model input), the last (**d**) the gas concentrations (model inputs).

**Figure 15:**
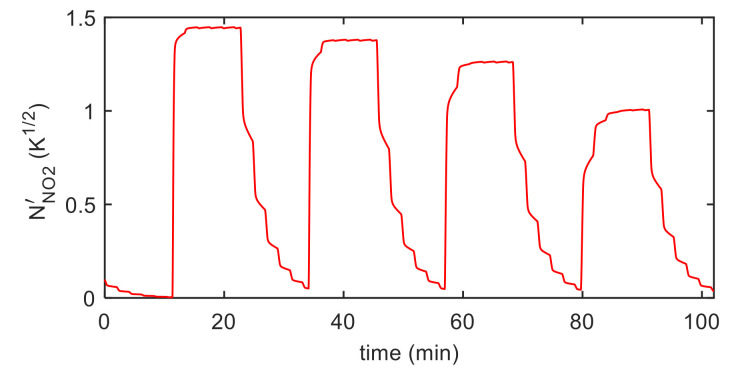
Results of the model fitting for an Au decorated MWCNT–COOH sensing film. Estimated chemisorbed NO_2_ as a function of time (model output).

Finally, the estimation of the necessary parameters ($k_{0NO2},\;\lambda_{NO2},\;k_{iNO2},\;\lambda_{iNO2},\;{\left[S\right]}^{\prime },\;T_{10},\;\nu \;,\;R_{B}\;)$ was obtained using measurements in air, N_2_ and NO_2_ mixtures with variable concentrations and variable temperature, as described before. Examples of the model fitting results are reported in Figure 12 and Figure 13 for the pristine MWCT–COOH sensor and in Figure 14 and Figure 15 for the Au functionalized sensing film.

Table 1 reports the estimated parameters values, obtained by model fitting.

The estimated conductances fit rather well the measured ones for both the tested materials, the fitting error was always below 5%. Therefore, the simplified model is able to catch the relevant features of the film response, showing the consistency of the considered assumptions.

The simple form of the model allows for understanding the dependence of the main sensing performance of the two materials under test on the main influence quantities.

In particular, it emerges that for these materials, the sensing film response is entirely due to the NO_2_ chemisorption, being the chemisorption of oxygen negligible.

Therefore:

(22) $$N_{s}^{\prime }\approx N\prime_{NO2}$$

and the adsorbed charge is described by the single reaction kinetics in Equation (23). It can be useful to rearrange that equation as follows:

(23) $$\frac{dN_{s}^{\prime }}{dt}=A_{NO_{2}}v_{f}-N_{s}^{\prime }/\tau \;$$

where $v_{f}=$ $k_{2}{\left[S\right]}^{\prime }e^{-\frac{\lambda_{NO}{}_{2}}{T}}\;$ is the rate of chemisorption at the reference NO_2_ concentration, and $A_{NO_{2}}v_{f}$ is the rate of chemisorption that depends only on temperature and gas concentration, whereas $\frac{N_{s}^{\prime }}{\tau }$, being $\tau ={\left(A_{NO_{2}}k_{2}e^{-\frac{\lambda_{NO}{}_{2}}{T}}+k_{0iNO_{2}}e^{-\frac{\lambda i_{NO}{}_{2}}{T}}\right)}^{-1},\;$ is the rate of desorption, which varies with temperature, with the target gas concentration and depends on the adsorbate surface concentration. As it can be seen from Equation (23), τ is a time, that depends on the temperature and gas concentration, it assumes the role of a time constant in steady working conditions and determines the response and recovery times. In detail, at fixed temperature the maximum value for the time constant is found when $A_{NO_{2}}=0$, i.e., with a NO_2_ concentration equal to 0, therefore the recovery time is always larger than the response time. On the other hand, the response time depends on the gas concentration: larger concentrations are characterized by shorter time constants.

When both the gas concentration and the temperature are constant the steady state value for *N_s_*′ is:

(24) $$N_{s}^{\prime }{}_{eq}=A_{NO_{2}}v_{f}\tau .$$

**Figure 16:**
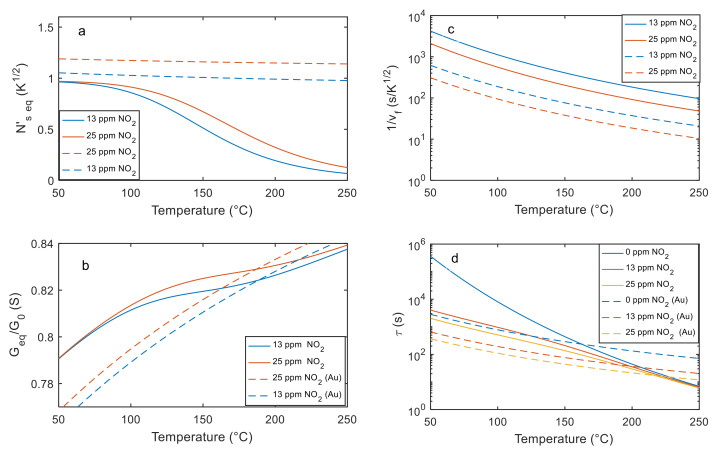
Steady state performance of the tested materials as a function of the working temperature: (**a**) estimated $N_{s}^{\prime }{}_{eq}$ at two different gas concentrations (Equation (24)); (**b**) normalized sensing film conductance (at the equilibrium); (**c**) inverse of the adsorption rate for two different gas concentrations; (**d**) time constants in the presence of two gas concentrations and in the absence of NO_2_ as per legend.

Obviously, due to the exponential relationship with temperature in the Arrhenius form of the reaction rate constants, the steady state value, the reaction rates and the time constants heavily depend on temperature, as can be seen for both the results reported in Figure 16 and in Table 2.

Using the estimated parameters to evaluate the equilibrium chemisorbed molecule surface density (Equation (24)), it can be found that it decreases with temperature, therefore the adsorption reaction is expected to be favored at low temperature, as can be observed in Figure 16.

Figure 16c,d shows the time constant behavior and the chemisorption rate.

The recovery time is about 2*τ* when $\tau =k_{0iNO_{2}}e^{-\frac{\lambda i_{NO}{}_{2}}{T}}$, and the dependence of this parameter on working temperature can be described by its relative sensitivity:

(25) $$\frac{d\tau }{dT}\frac{1}{\tau }=\frac{\lambda i_{NO}{}_{2}}{T^{2}}\;$$

which reduces with increasing temperature. For both tested materials, this parameter goes from 6% at 120 °C to 4% at 220 °C, showing a more critical situation at low temperature. Below 150 °C the recovery time of both the sensing materials becomes too large for any practical applications.

It can be seen from all the presented results that the addition of gold nanoparticles has mainly the effect of significantly reducing the time constant at low temperature, therefore allowing a reduction of the working temperature, and of increasing the amount of chemisorbed NO_2_, thereby improving the sensor response magnitude.

**Figure 17:**
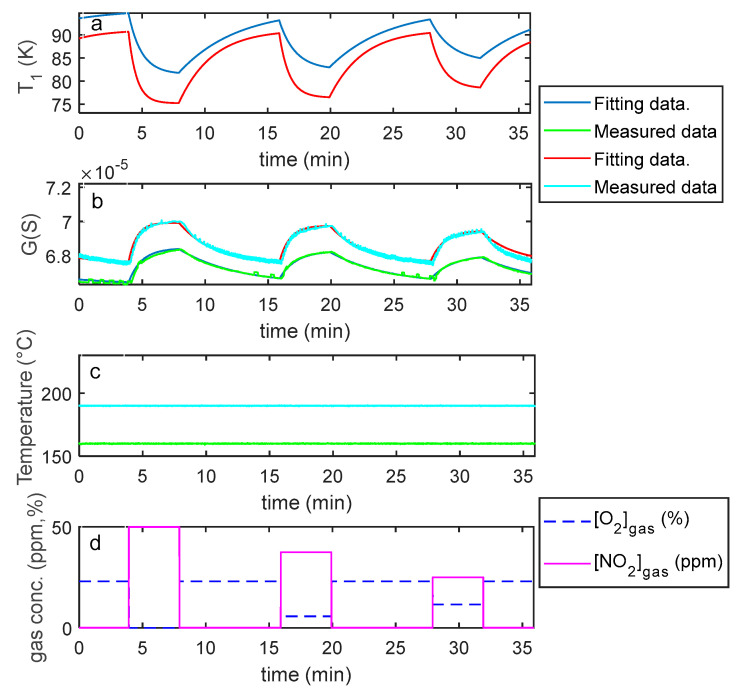
Comparison of experimental data and of the model predictions for an MWCNT–COOH sensing film. From the top: the first plot (**a**) shows the estimated *T*_1_ as a function of time (model output), the second plot (**b**) compares the estimated *G*(*t*) (model output) with the measured one as per legend, the third (**c**) shows the measured temperature (colors correspond to the measured *G*), the last (**d**) the gas concentrations.

**Figure 18:**
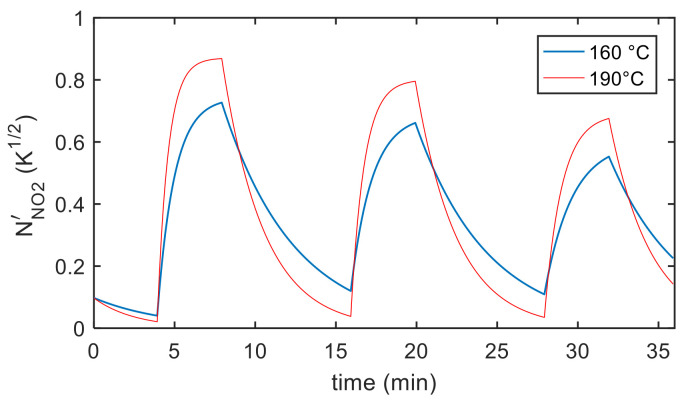
Predicted chemisorbed NO_2_ as a function of time for an MWCNT–COOH sensing film (corresponding to results shown in Figure 17).

**Figure 19:**
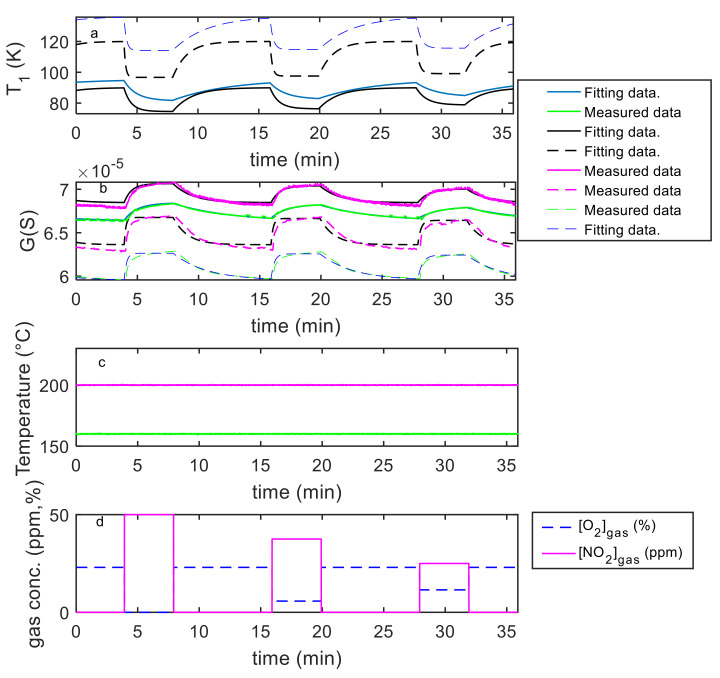
Comparison of experimental data and model predictions for an MWCNT–COOH sensing film (continuous lines) and for an MWCNT–COOH + Au film (dashed lines). From the top: the first plot (**a**) shows the estimated *T*_1_ as a function of time (model output), the second plot (**b**) compares the estimated *G*(*t*) (model output) with the measured one as per legend, the third (**c**) shows the measured temperature (colors correspond to the measured *G*), the last (**d**) the gas concentrations.

**Figure 20:**
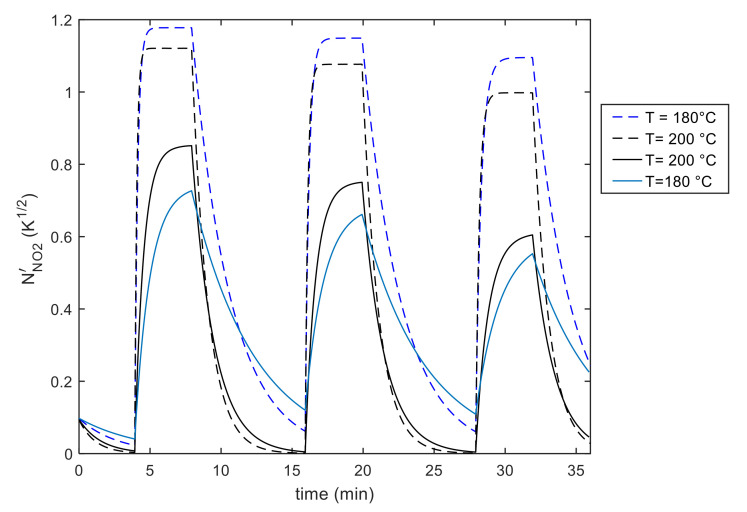
Predicted chemisorbed NO_2_ as a function of time for an MWCNT–COOH (continuous lines) and for an MWCNT–COOH + Au (dashed lines) sensing film (corresponding to results shown in Figure 19).

It is worth noting that the absorption and desorption rates largely vary in magnitude with temperature and gas concentration for the MWCNT–COOH film. In contrast, the variation with temperature of the time constants for the Au decorated film is reduced. Moreover, the addition of gold nanoparticles increases the response. The behavior predicted by this analysis is confirmed by observing the application of the model to constant temperature experiments such as those shown in Figure 17, Figure 18, Figure 19, Figure 20. It can be seen that the model can fit satisfactorily the experimental data and can be used to analyze the behavior at constant temperature and with variable NO_2_ concentration.

Already at 190 °C the pure MWCNT fails to recover entirely in 8 min, as expected.

It is worth noting that the estimated *T*_1_ is in the range 80 K–140 K for both materials, whereas ν has a small value indicating that the term $\nu T_{1}^{3/4}$ in the denominator of Equation (20) could have been neglected, confirming also results in the literature [34]. Moreover, the exponential term in the expression of conductance of Equation (20) has a magnitude in the range (0.2–0.4), showing that in the operating range the conductance of these materials could be approximated with a relative error lower than 10% by a linear relationship with the inverse of the temperature:

(26) $$G=\frac{1}{R_{B}}\;\left(1-\frac{T_{10}-2\sqrt{T_{10}}\;N_{S}^{\prime }+N_{S}^{\prime }{}^{2}}{T}\right)$$

Moreover, considering the estimated values for the parameter *T*_10_ and the estimated value for $N_{S}^{\prime }$, Equation (26) can be further simplified leading to the following result:

(27) $$G\approx \frac{1}{R_{B}}\;\left(1-\frac{T_{10}}{T}+\frac{2\sqrt{T_{10}}\;N_{S}^{\prime }}{T}\right)$$

being $2\sqrt{T_{10}}\;N_{S}^{\prime }\gg N_{S}^{\prime }{}^{2}$.

In case of steady state conditions, Equation (27) can be used to predict the sensor response as a function of gas concentration combining Equation (27) with Equation (24), as follows:

(28) $$G\approx \frac{1}{R_{B}}\;\left(1-\frac{T_{10}}{T}+\frac{2\sqrt{T_{10}}\;A_{NO_{2}}v_{f}\tau }{T}\right)$$

Therefore, the variation of the sensing film conductance due to NO_2_ adsorption can be written as follows:

(29) $$\Delta G\approx {\frac{2\sqrt{T_{10}}\;A_{NO_{2}}v_{f}}{T\left(A_{NO_{2}}k_{2}e^{-\frac{\lambda_{NO}{}_{2}}{T}}+k_{0iNO_{2}}e^{-\frac{\lambda i_{NO}{}_{2}}{T}}\right)}}^{}$$

showing that the relationship between the gas concentration and the conductance of the sensor, at fixed and sufficiently high temperature, can be approximated by a linear function for low gas concentration, when [*S*]′ >> *Ns*′.

A different behavior was found for SWCNT films for which the same type of thermally activated conduction model applies, but for which the exponential term was very large, always implying a highly non-linear relationship between conductance and gas concentration.

## 6. Conclusions

The paper shows that a simple model is suitable to describe the behavior of MWCNT networks as sensing materials for NO_2_ gas. This model can be used to unravel the mechanisms involved in sensing and as a basis for the development of gas sensors and monitoring systems based on these materials. The derived model simulates the film behavior and predicts the device responses in different operating conditions concerning the operating temperature and gas concentration. The model also analyzes the effect of functionalization with Au nanoparticles of the MWCNT based materials.

## Figures and Tables

**Table 1:** Estimated parameters for the two tested materials.

	MWCNT–COOH	MWCNT–COOH + Au
$k_{2}$ (s^−1^) (@ 25 ppm NO_2_)	9.4	18
$k_{0iNO_{2}}$ (s^−1^)	6.1 × 10^6^	7.06
$\lambda_{NO_{2}}$ (K)	3188	2752
$\lambda_{iNO_{2}}$ (K)	9182	2800
[*S*]′ (K^1/2^)	0.97	1.68
*T*_01_ (K)	95	114
*n* (K^1/4^)	0.3	0.04
*R_B_* (W)	12,145	11,944

**Table 2:** *v_f_* in different conditions (constant temperature and gas concentrations) for the two tested materials.

	MWCNT–COOH			MWCNT–COOH + Au		
	T = 120 °C	T = 150 °C	T = 200 °C	T = 120 °C	T = 150 °C	T = 200 °C
1/*v_f_* (@ 25 ppm NO_2_)	361 s/K^1/2^	200 s/K^1/2^	91 s/K^1/2^	9 s/K^1/2^	1.8 s/K^1/2^	3.2 s/K^1/2^
τ (@ 25 ppm NO_2_) RESPONSE	5.0 min	2.3 min	0.51 min	1.2 min	0.6 min	0.35 min
τ (@ 0 ppm NO_2_) RECOVERY	38 min	7.3 min	1.5 min	8.5 min	4.0 min	2 min
